# A simplified approach to disulfide connectivity prediction from protein sequences

**DOI:** 10.1186/1471-2105-9-20

**Published:** 2008-01-14

**Authors:** Marc Vincent, Andrea Passerini, Matthieu Labbé, Paolo Frasconi

**Affiliations:** 1 Machine Learning and Neural Networks Group, Dipartimento di Sistemi e Informatica, Università degli Studi di Firenze, Via di Santa Marta 3, 50139 Firenze, Italy

## Abstract

**Background:**

Prediction of disulfide bridges from protein sequences is useful for characterizing structural and functional properties of proteins. Several methods based on different machine learning algorithms have been applied to solve this problem and public domain prediction services exist. These methods are however still potentially subject to significant improvements both in terms of prediction accuracy and overall architectural complexity.

**Results:**

We introduce new methods for predicting disulfide bridges from protein sequences. The methods take advantage of two new decomposition kernels for measuring the similarity between protein sequences according to the amino acid environments around cysteines. Disulfide connectivity is predicted in two passes. First, a binary classifier is trained to predict whether a given protein chain has at least one intra-chain disulfide bridge. Second, a multiclass classifier (plemented by 1-nearest neighbor) is trained to predict connectivity patterns. The two passes can be easily cascaded to obtain connectivity prediction from sequence alone. We report an extensive experimental comparison on several data sets that have been previously employed in the literature to assess the accuracy of cysteine bonding state and disulfide connectivity predictors.

**Conclusion:**

We reach state-of-the-art results on bonding state prediction with a simple method that classifies chains rather than individual residues. The prediction accuracy reached by our connectivity prediction method compares favorably with respect to all but the most complex other approaches. On the other hand, our method does not need any model selection or hyperparameter tuning, a property that makes it less prone to overfitting and prediction accuracy overestimation.

## Background

Some interesting structural, functional, and evolutionary properties of proteins can be inferred from knowledge about the existence and the precise location of disulfide bridges. Since most of the proteins inferred from genomic sequencing lack this structural information, the ab-initio prediction of disulfide bridges from protein sequences can be very useful in several molecular biology studies. This computational problem has received significant attention during the last few years and a number of prediction servers have been recently developed [1-5].

Typical approaches predict disulfide bridges by solving two separate sub-problems. First, cysteines are partitioned into two groups: half-cystine (involved in the formation of a disulfide bridge) and the rest (free and bound to either a metal ion or to a prosthetic group). Membership in one of the two groups is predicted by a binary classifier trained on known cases. Second, given known bonding state, disulfide bridges are assigned by predicting which pairs of half-cystines are linked. The latter sub-problem is considerably more difficult from a machine learning perspective as it requires methods capable of predicting structured outputs. A noticeable exception to the two-stage approach was recently proposed in [6]. The main novelty in that method is the use of a recursive neural network that can predict the bonding probability between any pair of cysteines, so that bridges can be predicted *directly *from sequence (without previous knowledge of cysteine bonding state).

Methods for solving the bonding state sub-problem have been developed after Fiser *et al*. [7] first noted that amino acid composition around cysteines could predict their disulfide-bonding state. Neural networks were applied later by Fariselli *et al*. [8] and by Fiser & Simon [9], using multiple alignment profiles in a window centered around the target cysteine. Mucchielli-Giorgi *et al*. [10] introduced the idea of adding a global descriptor (consisting of amino acid composition and chain length information) to improve prediction accuracy. Ceroni *et al*. [11] proposed a method based on string kernels (to extract global features), vector kernels for handling local features, and supervised learning by support vector machines (SVM). The subsequent improvement described in [5] uses recurrent neural networks and Viterbi decoding to convert single predictions into a collective bonding state assignment for all the cysteines in a chain. Song *et al*. [12] applied a linear discriminant using dipeptides as features. Martelli *et al*. [13] suggested the use of hidden Markov models to refine local predictions obtained via neural networks. Chen *et al*. [14] used an ensemble of Support Vector Machines trained with different feature vectors refined by a linear-chain Markov model. In essence, state-of-the-art methods start by predicting the bonding state of each cysteine and then use a refinement procedure to improve chain prediction. Concerning bonding state prediction, in this paper, we show that (1) a much simpler technique based on binary classification of chains allows us to achieve the same levels of cysteine level accuracy, and (2) prediction errors obtained in this way do not completely overlap with those of our previous method (DISULFIND [5]) leaving room for accuracy improvement by exploiting a combination of the two classifiers.

The disulfide connectivity sub-problem was pioneered in [1] with a method based on weighted graph matching. Vullo & Frasconi [15] introduced the use of multiple alignment profiles by means of recursive neural networks (RNN). Taskar *et al*. [16] also formulated disulfide connectivity as a structured-output prediction problem and solved it using a generalized large-margin machine. Ferrè & Clote [17] proposed a feedforward neural-network architecture with hidden units associated to cysteine pairs and inputs encoding secondary structure. They recently extended their method to three-class discrimination (free, half-cystine, or metal bound) [2]. Tsai *et al*. [3] confirmed that the profile of distances between bonded cysteines is an important feature for prediction of connectivity patterns and devised a prediction method based on SVM and weighted graph matching. Zhao *et al*. [18] observed that the number of observed connectivity patterns is relatively small compared to the number of possible patterns: while a set of 2*B *cysteines can be potentially arranged in (2*B *- 1)!! = (2*B *- 1)·(2*B *- 3)···3·1 ways, only a few dozens of patterns are actually observed. Based on this observation they suggested a template-matching approach. A multiclass SVM was applied by Chen and Hwang [19] by considering each connectivity pattern as a distinct class. Finally, few recent works proposed more complex architectures. The method by Chen *et al*. [20] is based on a two-level strategy where all cysteine pairs are first classified by an SVM and all possible connectivity patterns are subsequently evaluated by second binary classifier that was trained with the correct connectivity patterns as positive examples. Lu *et al*. [21] proposed an ensemble of SVMs, using features derived from cysteine-cysteine coupling, and a genetic algorithm for feature selection; their method outputs the pattern maximizing the number of predicted pairwise interactions. In this paper, we show that a simple 1-nearest neighbor classifier considering both separation and evolutionary profiles is competitive to all previously proposed approaches, including those based on structured output prediction, with the exception of the most complex multiple stage architectures. The method needs no hyperparameter tuning, an appealing property making it more robust to overtraining. Model selection is in fact sometimes difficult to carry out and different choices of hyperparameters (e.g. a set of regularization coefficients in a multi-stage architecture) may affect significantly the results obtained in the experiments [22]. We hope in this way to provide a method which is less prone to overfitting and instabilities in the estimation of the generalization error.

### Overview of the proposed methods

We begin by observing that a significant fraction of chains tend to exhibit one of two extreme behaviors with respect to disulfide bridge formation: either no cysteines are bonded, or (almost) all of them are. Fiser & Simon [9] exploited this fact and showed that a majority voting scheme (by which the same neural network prediction is extended to all cysteines in a chain based on the majority of individual predictions) was able to improve prediction accuracy. Statistics on two data sets (see Data Preparation in Results and Discussion) are shown in Table 1. This justifies methods that focus on chain rather than cysteine classification. For example, if we classify all cysteines in a chain as disulfide bonded if and only if the chain is predicted to have at least one bridge, we incur in a predictive accuracy penalty of 3% and 6% for the PDBselect and SPX^- ^data set, respectively.

**Table 1:** Statistics of data sets

Data set	# chains	All	None	Mix
PDBselect	1,589	488	1,051	50
SPX^-^	2,547	1,650	757	140

Statistics for the PDBselect and the SPX^- ^data sets. The three types of chains are defined as follows. All: all cysteines are intra-chain bonded half-cystines. None: all cysteines are either free, metal bound, or inter-chain bonded. Mix: Both cases are present.

The new procedure for obtaining bridge predictions can be shortly summarized as follows (see Methods for details). In the first step, a kernel machine is trained to predict if a given chain contains at least one intra-chain bridge. For this task, a chain is represented as a bag of cysteines. The resulting decomposition kernel between two chains is the sum of all the similarities between the amino acid environments around all possible pairs formed by taking one cysteine from each chain. The rationale of this kernel is that a new chain should be similar to a positive chain if it contains at least one pair of cysteine environments which is similar to a pair that is known to form an intra-chain bridge. This kernel is called the all-pairs decomposition kernel (APDK) in the remainder of the paper. The experiments reported below are based on this kernel in conjunction with SVM. For this purpose, we employed the publicly available software SVM^*light *^[23].

In the second step, a set of kernel machines classify chains according to their connectivity pattern. Each of these machines focuses on a given number of cysteines. In this case, a chain is seen as a tuple of amino acid environments around its cysteines. The resulting decomposition kernel between two chains is the sum of the similarities between the environments associated with the two cysteines that have the same ordinal number in the tuple. The rationale of this kernel is that two chains should be more likely to fold according to the same disulfide connectivity pattern if they share a similar sequence of cysteine environments. This kernel is called the tuple decomposition kernel (TDK) in the remainder of the paper. The experiments reported in the next section use this kernel in conjunction with the cysteine separation profiles [3] to compute distances (in feature space) for the 1-nearest neighbor (1-NN) algorithm.

In both the above kernels, the amino acid environments around cysteines are enriched with evolutionary information derived from multiple alignments in order to boost performance.

## Results and discussion

### Data preparation

We used three representative subsets of the Protein Data Bank to assess the performance of our kernel methods. A third data set, extracted from the SWISS-PROT database, was employed in connectivity prediction assuming knowledge of the cysteine bonding state, for the sake of comparing results with respect to previous methods.

#### PDBselect data set

The July 2005 PDBselect data set [24,25] used in this paper contains 2,810 non redundant chains. During the chain selection process, for any group of chains with homologies, only the one with the best quality was kept. The structures of chains included were determined both using NMR and X-ray crystallography. See [26] for the complete list of chains with explanations. Disulfide bridges were obtained by running the DSSP program [27] with default options. Unresolved residues were labeled as free. In order to reduce noise in the data, we visually inspected protein structures in all cases in which two cysteines were found within a distance of 2.5 Å, but were not labeled by DSSP as being disulfide bonded. In 62 cases we over-ruled the DSSP assignment from free to disulfide bonding. For the chain classification experiments only proteins with at least 2 cysteines were considered, resulting in a set of 1,589 chains. The final data set with labeling information is available as Additional file 1.

#### SPX data set

The data set is described in [6] and available from the DIpro website [28]. It consists of two sets of chains: one used for the chain classification problem (SPX^-^), the other used for the pattern classification problem (SPX^+^). The former contains 897 chains with at least one intra-chain bridge (positive examples) and 1,650 without any intra-chain bridge (negative examples), for a total of 2,547 chains. The latter contains 1,018 chains with at least one intra-chain bridge. Positives chains in SPX^- ^are less redundant (HSSP cutoff of 5) than those in SPX^+^(HSSP cutoff of 10). A first difference with respect to the PDBselect data set is that no chain in the SPX data set contains inter-chain disulfide bonds. A second difference is that disulfide bonds in SPX are extracted from the SSBOND record of the PDB files [6].

#### PDB_4136_

The data set is described in [13] and available from the CysPred website [29]. It consists of 4,136 cysteine containing segments from the crystallographic data of the PDB, with less than 25% sequence identity and no chain breaks. The data set is included for the sake of comparison with the approach by Chen *et al*. [14].

#### SP39 data set

The data set is described in [1,15] and available as Additional file 2. It consists of 446 chains from the SWISS-PROT database release n. 39 (October 2000), having from two to five experimentally verified intra-chain disulfide bridges. The data set has been widely used as a benchmark for disulfide connectivity prediction assuming knowledge of the cysteine bonding state.

In order to incorporate evolutionary information, we obtained multiple alignments by running one iteration of the PSI-BLAST [30] program on the non-redundant (nr) NCBI database using an E-value cutoff of 0.005. Depending on the experimental setting, we have used either position specific scoring matrices (PSSM) or multiple alignment profiles.

### Evaluation procedure

#### Bonding state

Prediction performance was estimated by a 10-fold cross-validation procedure for PDBselect and SPX, while for PDB_4136 _we employed a 20-fold cross validation procedure with exactly the same folds as in [13]. For each of the folds, we optimized the main hyperparameters (i.e. the kernel Gaussian width *γ*, and the SVM regularization parameter *C*) by nesting a 10-fold cross-validation on each training set.

Hyperparameters were found by a variable-resolution grid search algorithm in which we started by optimizing on a coarse log-scale and then refined the best set of hyperparameters on a finer scale. In this setting, a significant computational speed-up was obtained by caching the entire kernel matrix in memory.

#### Connectivity patterns

Prediction performance was estimated by a 10-fold cross-validation procedure for the SPX^+ ^and SPX^- ^data sets, while for the SP39 data set we employed a 4-fold cross validation procedure with exactly the same folds as in [1,15]. No model selection was carried out to fine tune kernel parameters, as our aim was to show the predictive power of the plainest approach.

### Performance measures

For binary classification problems, let us denote by *T*_*p*_, *T*_*n*_, *F*_*p*_, and *F*_*n *_the number of true positives, true negatives, false positives, and false negatives, respectively. Also let *N *denote the total number of cases. We report the following measures:

**accuracy ***Q *= (*T*_*p *_+ *T*_*n*_)/*N*;

**precision ***P *= *T*_*p*_/(*T*_*p *_+ *F*_*p*_);

**recall ***R *= *T*_*p*_/(*T*_*p *_+ *F*_*n*_).

In the case of bonding state predictions we can define the above measures at different levels:

• Cysteine classification measures: *Q*_*c*_, *P*_*p*_, *R*_*p*_. These are obtained by counting single cysteines as cases.

• Sequence classification measures: *Q*_1_, *P*_1_, *R*_1_. These are obtained by counting chains as cases. Positive examples are chains having at least one intra-chain bridge and negative examples are all the remaining chains.

Performance measures for the disulfide pattern prediction problems are defined as follows:

• Pattern prediction accuracy: *Q*_*p *_defined as the total number of chains for which the correct pattern was predicted, divided by the total number of chains.

• Bridge-level precision *P*_*b*_, defined as the number of correctly predicted bridges divided by the number of predicted bridges, and bridge-level recall *R*_*b*_, defined as the number of correctly predicted bridges divided by the true number of bridges.

### Binary classification of chains and cysteines

Table 2 reports the 10-fold cross-validation results for the all-pairs decomposition kernel (see Equation 2 in Methods) applied to both the PDBselect and SPX^- ^data set. Positive examples are chains with at least one intra-chain bridge. Results are reported using both PSSM and profiles. One goal of these experiments is to assess the effectiveness of the chain-level APDK and compare it to the more traditional approach in which the classifier is based on a kernel defined at the cysteine-level. For this purpose, we retrained the state-of-the-art cysteine bonding state predictor DISULFIND [5] using the same 10-fold cross-validation procedure, classifying a chain as positive if at least one cysteine was predicted to be bound. Since DISULFIND uses a rather sophisticated procedure to post-process predictions on single cysteines, we also trained a simplified version of DISULFIND (D-simple) based on single cysteine classification, without post-processing. Chain-level predictions were obtained from D-simple predictions by majority voting (we found this scheme outperformed the rule based on logical OR).

**Table 2:** Binary classification of chains

Method	PDBselect			SPX^-^		
	*Q*_1_	*P*_1_	*R*_1_	*Q*_1_	*P*_1_	*R*_1_
APTK (PSSM)	87	83	77	82	79	67
APTK (profiles)	86	83	75	82	80	64
DISULFIND (PSSM)	86	82	75	81	80	60
DISULFIND (profiles)	86	82	73	81	80	63
D-simple (PSSM)	85	81	74	82	81	64
D-simple (profiles)	86	84	74	82	82	62
DIpro [6]	-	-	-	74	83	56

Experimental comparison of various algorithms for binary classification of chains. A chain is a positive examples if and only if it has least one intra-chain disulfide bridge. We report two-state prediction accuracy (data sets. *Q*_1_), precision (*P*_1_) and recall (*R*_1_) on both PDBselect and the SPX^- ^data sets.

The last row contains the best results reported in [6] for the DIpro predictor on the SPX^- ^data set, obtained with a spectrum kernel with mismatches.

The protein chain classification approach can also obtain a good predictive accuracy at the level of single cysteine bonding state. APTK results in Table 3 were obtained by classifying all cysteines in a chain as a half-cystines if they belong to a chain that has been predicted to have at least one intra-chain bridge. Although this approach implies a systematic error on chains of mix type (i.e. those containing both free cysteines and half-cystines) these cases are not frequent and the overall accuracy is comparable to that of DISULFIND, as shown in Table 3. Note that the APTK on chains outperforms D-simple without the need for a complex architectural design. This shows that the chain-level kernel yields better results than the cysteine-level kernel even when the task is the classification of individual cysteines.

**Table 3:** Binary classification of cysteines

Method	PDBselect			SPX^-^			PDB_4136_		
	*Q*_*c*_	*P*_*c*_	*R*_*c*_	*Q*_*c*_	*P*_*c*_	*R*_*c*_	*Q*_*c*_	*P*_*c*_	*R*_*c*_
APTK (PSSM)	88.8	83.8	87.7	86.1	79.0	82.8	88.2	84.0	82.5
APTK (profiles)	87.7	83.1	85.1	85.3	78.6	80.5	89.7	81.0	88.5
DISULFIND (PSSM)	88.3	85.0	84.3	85.3	82.6	74.1	88.0	79.1	85.5
DISULFIND (profiles)	88.6	87.4	82.1	86.5	83.0	77.5	89.4	81.2	87.4
D-simple (PSSM)	82.2	77.0	76.4	81.3	74.5	71.5	83.0	79.5	69.3
D-simple (profiles)	81.5	76.0	75.3	81.1	74.3	71.1	83.0	77.1	73.4
APTK + DISULFIND	89.9	87.8	85.5	87.0	82.6	80.2	90.3	82.1	89.2
multiple SVM + CSS [14]	-	-	-	-	-	-	90	91	77

Experimental comparison of various algorithms for binary classification of cysteines. Positive examples are disulfide-bond cysteines. We report two-state prediction accuracy (*Q*_*c*_), precision (*P*_*c*_) and recall (*R*_*c*_) on PDBselect, SPX^- ^and PDB_4136 _data sets.

What is perhaps more interesting, is that a correlation analysis between the errors of the APTK and DISULFIND reveals that the two predictors disagree on many of the cysteines that are incorrectly classified by one of the two methods (10.6% of such cysteines on PDBselect and 8.6% on SPX^-^). The relatively low correlation between the two methods may be an advantage because we can hopefully boost performance by combining their predictions. Indeed, as reported in the last row of Table 3, by combining APTK (with PSSM) and DISULFIND (with profiles) we gain about one percentage point of accuracy in both data sets. The last columns of Table 3 report evaluation results on the PDB_4136 _data set, which confirm the overall behavior, with the exception that profiles are always as good or better than PSSM, and are thus employed in the APTK + DISULFIND combination. The combination achieves basically the same accuracy as that of the best method from Chen *et al*. [14], which we indicated by multiple SVM + CSS, and shows a more balanced precision/recall ratio.

### Connectivity prediction for positive chains

Table 4 reports connectivity prediction results for the SPX^+ ^data set. In this experiment, chains are known to have at least one intra-chain bridge. The proposed 1-nearest neighbor (1-NN) classifier is compared to DISULFIND and to DIpro (results published in [6]). In spite of its simplicity, 1-NN outperforms these methods, both at the level of individual bridge prediction and at the level of whole connectivity pattern prediction.

**Table 4:** Prediction of bridges and connectivity patterns

# bridges	1-NN			DISULFIND			DISULFIND+1-NN			DIpro		
	*R*_*b*_	*P*_*b*_	*Q*_*p*_	*R*_*b*_	*P*_*b*_	*Q*_*p*_	*R*_*b*_	*P*_*b*_	*Q*_*p*_	*R*_*b*_	*P*_*b*_	*Q*_*p*_
1	65	61	58	66	62	59	68	63	59	71	47	58
2	59	61	52	53	54	49	68	69	63	59	59	55
3	70	71	63	46	46	35	73	73	64	59	65	50
4	58	59	42	24	24	9	59	59	48	44	49	27
all	60	59	52	49	48	41	64	62	55	71	47	48

Chains with at least one intra-chain bridge (SPX^+ ^data set): comparison between 1-NN, DISULFIND, DISULFIND+1-NN and DIpro.

By inspecting the overall results (all number of bridges, last row of Table 4) we note that precision and recall levels for bridge prediction are very similar for both 1-NN and DISULFIND. Conversely, DIpro has higher recall but lower precision, which is mainly to be due to the higher number of false positives in the case of chains with a single bridge.

Note that the bonding state of individual cysteines is unknown in the SPX^+ ^data set. Thus the 1-NN algorithm is also implicitly solving a bonding state prediction problem (although it was not expressly tuned for this purpose). Alternatively, the 1-NN classifier can be preceded by an explicit bonding-state predictor. In this case, the tuple-based kernel (see Equation 5 in Methods) as well as the topological features are restricted to those cysteines that are known to be bonded (for training examples) or that are predicted to be bonded (for test examples) when computing the nearest neighbor. We employed the first stage of DISULFIND to this aim, and the results of such a pipeline are reported in the DISULFIND+1-NN column of Table 4. The advantage of this combination is more evident in the case of two and four bridges.

### Connectivity prediction assuming knowledge of the bonding state

The performance of connectivity prediction methods is often assessed independently of the performance of an underlying bonding state predictor. In order to compare our approach to the literature state-of-the-art, we carried out experiments on the SP39 data set, assuming the bonding state was known a priori. Results are reported in Table 5. In this setting, the number of predicted bridges and the number of true bridges are necessarily the same and, therefore, *P*_*b *_= *R*_*b*_. We also took advantage of bonding state knowledge by slightly changing the TDK that defines distances for the 1-NN classifier to use only bonded cysteines in the summation of Equation 5 (see Methods).

**Table 5:** Prediction of bridges and connectivity patterns

# bridges	1-NN		DISULFIND		DIpro		SOSVM		CSP		SVMpattern	
	*P*_*b *_= *R*_*b*_	*Q*_*p*_	*P*_*b *_= *R*_*b*_	*Q*_*p*_	*P*_*b *_= *R*_*b*_	*Q*_*p*_	*P*_*b *_= *R*_*b*_	*Q*_*p*_	*P*_*b *_= *R*_*b*_	*Q*_*p*_	*P*_*b *_= *R*_*b*_	*Q*_*p*_
2	76	76	73	73	74	74	77	77	73	73	74	74
3	66	55	51	41	61	51	62	52	66	55	69	61
4	53	38	37	24	44	27	51	36	49	33	40	30
5	39	18	30	13	41	11	43	13	36	17	31	12
2–5	64	55	49	44	56	49	65	53	62	53	57	55

Results obtained assuming knowledge of the bonding state (SP39 data set): comparison between 1-NN, DISULFIND, DIpro, SOSVM, CSP and SVMpattern.

Results from 1-NN are compared to those found in the literature for the following single-stage approaches: DISULFIND [15], DIpro [6], Taskar et al.'s structured output large margin algorithm [16] (SOSVM), the pattern-wise SVM by Chen and Hwang [19] (SVMpattern), while the cysteine separation profile approach (CSP) by Tsai *et al*. [3] was reimplemented in order to get results on exactly the same folds. In predicting entire connectivity pattern (*Q*_*P*_), 1-NN outperforms all other methods except SVMpattern that obtains the same overall results. On the other hand, SVMpattern is worse at predicting single pairwise interactions (*Q*_*c*_), where SOSVM is the only approach achieving slightly better results. The latter, however, requires solving a hard convex optimization problem. It is interesting to note that the accuracy of 1-NN is consistently better than that of all other methods for more than three bridges. This is quite reasonable, as increasing the number of bridges, *B*, implies dramatically increasing the number of alternative patterns, (2*B *- 1)!!, while lowering the amount of available data as well as the number of observed patterns (see Prediction of Connectivity Patterns in Methods). Such a setting favors 1-NN, which can only predict observed patterns. The small advantage of 1-NN with respect to CSP is due to the contribution of the evolutionary profile to the distance metric. In order to elucidate the cases in which this advantage is apparent, we analysed the differences between chains incorrectly predicted by the two methods. The analysis showed that the main reason for the increase in performance of 1-NN is due to correctly predicting the pattern of chains from two families: the Alpha-type family in the conotoxin A superfamily, and the Alpha subfamily of the Sodium channel inibitor family, within the long (4 C-C) scorpion toxin superfamily. Note however that in few cases adding the evolutionary profile actually decreased performance with respect to CSP, as happened for two chains from the glycosyl hydrolase 13 family.

Some recent multi-stage architectures [20,21] outperform the above mentioned methods. However, the aim here is to stress the effectiveness of our method in comparison to the other existing single-stage approaches. By requiring no hyperparameter tuning, it can also be seen as a candidate component that might be more easily integrated into complex architectures.

### Connectivity prediction from scratch

We finally evaluated the performance of the method in predicting connectivity from scratch, that is assuming no knowledge about disulfide bonding state for a chain. The APTK predictor is employed to predict whether a given chain has at least one intra-chain bridge, while the 1-NN predicts the connectivity pattern for chains predicted to be positive. Table 6 reports experimental results on the SPX^- ^data set for the APTK(PSSM)+1-NN pipeline, compared to DISULFIND and to the results for DIpro as reported in [6], confirming the advantage of the proposed architecture. The only case in which DISULFIND slightly outperforms APTK(PSSM)+1-NN is when chains have a single bridge. Clearly in such a case the pattern prediction problem boils down to that of correctly detecting the two bonded cysteines. The DISULFIND connectivity predictor is not involved in this case, and 1-NN is only competing against the first stage of DISULFIND, which is explicitly trained for bonding state prediction. The DISULFIND+1-NN pipeline combines the advantage of the state-of-the-art bonding state predictions of DISULFIND with that of the 1-NN, achieving the best performance both overall and for most bridge numbers.

**Table 6:** Prediction of bridges and connectivity patterns from scratch

# bridges	APTK(PSSM)+1-NN			DISULFIND			DISULFIND+1-NN			DIpro		
	*R*_*b*_	*P*_*b*_	*Q*_*p*_	*R*_*b*_	*P*_*b*_	*Q*_*p*_	*R*_*b*_	*P*_*b*_	*Q*_*p*_	*R*_*b*_	*P*_*b*_	*Q*_*p*_
1	30	30	27	30	30	30	30	30	30	-	-	-
2	51	54	47	38	39	36	51	51	49	-	-	-
3	63	65	58	27	27	15	66	67	61	-	-	-
4	50	51	40	30	30	10	48	49	37	-	-	-
all	43	44	37	29	29	23	43	44	39	32	48	-

Results on the SPX^- ^data set: comparison between APTK (PSSM)+1-NN, DISULFIND, DISULFIND+1-NN and DIpro.

## Conclusion

We have presented a new set of kernel-based methods for predicting disulfide bridges and cysteine bonding state from protein sequences. Despite their extreme simplicity, these algorithms compare favorably to most existing techniques proposed in the literature. In the case of cysteine bonding state we have found that the correlation between predictions from the new chain classifier and previous methods (DISULFIND) is low enough to allow us improving accuracy by combining the two classifiers. The combination achieves competitive results with the state-of-the-art approaches. Concerning connectivity pattern prediction, we found that a simple 1-nearest neighbor approach performs surprisingly well, being outperformed by the most complex multi-stage architectures only. It must be remarked that the algorithm does not need any hyperparameter tuning, which makes it less prone to overfitting and prediction accuracy overestimation, and appealing for prediction of other properties of proteins that are inherently structured. The result is also interesting from a machine learning perspective as it shows that, depending on the probability distribution on the output space, it may be an advantage to employ multiclass classification instead of much more complex algorithms for prediction of structured outputs. Different approaches to classification could be used in place of 1-NN. For example, using multiclass support vector machines one could take advantage of a loss function that weights differently prediction errors according to the number of correctly assigned bridges. These variants are currently under our investigation.

## Methods

### Decomposition kernels

A kernel is a real-valued function *K *: X × X ↦ ℝ where X is the input space and can be any set (e.g., a set of protein chains). If for any finite set of instances {*x*_1_,....,*x*_*m*_}, *x*_*i *_∈ X the matrix with entries *K*(*x*_*i*_, *x*_*j*_) has all non-negative eigenvalues, then *K *is *positive semi-definite *and by Mercer's theorem there exists a *feature space *H and a map *φ *: X ↦ H such that the kernel can be written as the inner product in feature space: *K*(*x*, *x'*) = ⟨*φ*(*x*), *φ*(*x'*)⟩.

In this paper, X is typically a set of protein chains. We propose to represent each chain as a structured object. Such a representation suggests the use of decomposition (or convolution) kernels [31], a vast class of functions that rely on two main concepts: (1) decomposition in parts of the structured objects and (2) composition of kernels between these parts. In this case, for *x *∈ X, suppose (*x*_1_,...,*x*_*D*_) is a tuple of *parts *of *x*, with *x*_*d *_∈ $X_{d}$ for each part type *d *= 1,...,*D*, which can be formally expressed by a decomposition relation *R*(*x*_1_,...,*x*_*D*_, *x*). Assuming that for each part type a Mercer kernel *κ*_*d *_: $X_{d}$ × $X_{d}$ ↦ ℝ is available, the decomposition kernel between two structured objects *x *and *x' *is defined as

(1) $$K(x,x^{\prime })=\sum_{\begin{array}{l} \left(x_{1},...,x_{D})\in R^{-1}(x\right)\\ \left({x^{\prime }}_{1},...,{x^{\prime }}_{D})\in R^{-1}(x^{\prime }\right) \end{array}}\prod_{d=1}^{D}K_{d}(x_{d},{x^{\prime }}_{d})$$

where *R*^-1^(*x*) = {(*x*_1_,...,*x*_*D*_) : R(*x*_1_,...,*x*_*D*_, *x*)} denote the set of all possible decompositions of *x*. In the following, we introduce new decomposition kernels for protein chains.

### Bonding state prediction

Let us decompose chains using as parts cysteines and their amino acid environments. Specifically, let us assume *D *= 1 and let *R*(*c, x*) hold true if *c *is cysteine in *x*. In this way, *R*^-1^(*x*) = cys(*x*), the set of cysteines in *x*.

In addition, we incorporate evolutionary information by using multiple alignments. Specifically, for a given cysteine *c*, let *s*(*c*) denote the (2*k *+ 1) × 20 matrix encoding the evolutionary information in a context of 2*k *+ 1 residues centered around *c*. We first introduce a kernel function on pairs *κ *of matrices: *κ *(*s*(*c*), *s*(*c'*)) = ⟨*s*(*c*), *s*(*c'*)⟩_*F *_where ⟨·,·⟩_*F *_denotes the Frobenius product between two matrices, which is defined for two *R *× *C *matrices *M *× *N *as:

$${\langle M,N\rangle }_{F}=\sum_{r=1}^{R}\sum_{c=1}^{C}M[r,c]N[r,c]$$

The all-pairs decomposition kernel (APDK) is then defined as follows:

(2) $$K_{C}(x,x^{\prime })=\sum_{c\in \text{cys}(x)}\sum_{c^{\prime }\in \text{cys}(x^{\prime })}\kappa (s(c),s(c^{\prime }))$$

The kernel function obtained in this way is thus based on pairwise comparisons between all cysteines in two given chains. Since the sum can be interpreted as a kind of soft OR operator, the intuitive meaning of Equation 2 is that two chains are dissimilar if no cysteine in one chain has a conserved amino acid environment that is similar to that of another cysteine in the other chain.

Composition with other kernels (in particular, the Gaussian kernel) can be used to obtain more complex decision functions. Two possibilities are allowed: the first, which we will refer to as the inner Gaussian, applies function composition on *κ*; the second, which we will refer to as the outer Gaussian, applies function composition on *K*_*C*_. Preliminary experiments showed that the inner Gaussian composition slightly outperforms the outer Gaussian composition. Hence the latter was never used in the experiments reported in this paper. The resulting kernel should also be normalized in order to avoid large matches due simply to a high number of cysteines.

(3) $${\bar{K}}_{C}(x,x^{\prime })=\frac{K_{C}(x,x^{\prime })}{\sqrt{K_{C}(x,x)K_{C}(x^{\prime },x^{\prime })}}.$$

### Combination between the all-pairs kernel and DISULFIND

We describe here the strategy used to obtain a weighted majority voting algorithm based on the predictions from DISULFIND [5] and from the APTK. DISULFIND outputs a conditional probability *p *while the APTK a real-valued margin in (-∞ +∞). In order to combine the two classifiers, we first convert DISULFIND output into a pseudo-margin $f_{d}=\log\frac{p}{1-p}$. This operation can be interpreted as the inverse of converting margins into conditional probabilities by using a sigmoidal function as described in [32]. The margin from the APDK, *f*, and the DISULFIND pseudo-margin, *f*_*d *_are then summed and the prediction about the bonding state of a cysteine is obtained by taking the sign of the result. Note that the margin *f *depends on the entire chain while the pseudo-margin *f*_*d *_depends on the particular cysteine. Thus, combined predictions are cysteine-specific.

### Prediction of connectivity patterns

Suppose that at least one disulfide bridge is known to exists for a given protein chain. In this case, if the chain contains *n *cysteines, the number of alternative disulfide connectivity patterns can be computed as

(4) $$D(n)=\sum_{B=1}^{\left\lfloor \frac{n}{2}\right\rfloor }\left(\begin{array}{c} n\\ 2B \end{array}\right)(2B-1)!!$$

The above formula can be explained as follows. Assuming the number of bridges is *B*, there are (2*B *- 1)!! alternative patterns. The number of bridges can vary from 1 to $\left\lfloor \frac{n}{2}\right\rfloor$ and for each case, there are $\left(\begin{array}{c} n\\ 2B \end{array}\right)$ possible subsets of cysteines that form these bridges.

**Figure 1:**
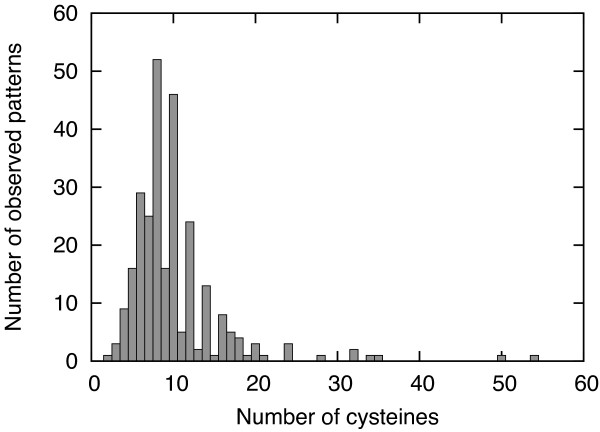
**Observed connectivity patterns**. Number of observed distinct patterns on the SPX^+ ^data set, grouped by number of cysteines.

**Figure 2:**
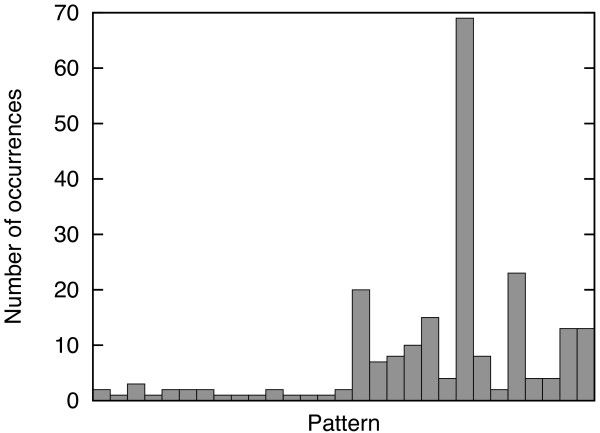
**Observed frequencies on chains having 6 cysteines**. Histogram of the number of occurrences of distinct patterns for chain having 6 cysteines on the SPX^+ ^data set. Patterns are sorted by rank.

**Figure 3:**
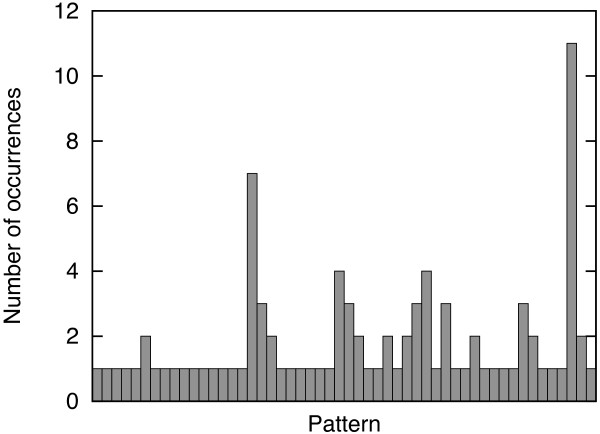
**Observed frequencies on chains having 8 cysteines**. Histogram of the number of occurrences of distinct patterns for chain having 8 cysteines on the SPX^+ ^data set. Patterns are sorted by rank.

In practice, the number of *observed *connectivity patterns is significantly smaller than *D*(*n*). In Figure 1 we report the number of observed patterns for the SPX^+ ^data set (see Data Preparation in Results and Methods), for different numbers of cysteines. For example for *n *= 8 only 52 out of 763 possible patterns are observed and for *n *= 10 only 46 out of 9,495 possible patterns. Of course, chains with a large number of cysteines are rarer but this alone does not explain these findings because the distribution over the patterns that are observed is also rather skewed. For example, we show the histogram of occurrences of different patterns for *n *= 6 and *n *= 8 in Figures 2 and 3, respectively.

The above analyses suggest that connectivity patterns may be predicted by defining a set of multiclass classification problems (one for each value of *n*) where the class corresponds to the pattern. Although the number of classes can be high for many values of *n*, experimental results show that this approach can be successfully pursued.

In order to solve the multiclass classification problem we introduce a decomposition kernel on chains based on the ordered *tuple *of cysteines (*c*_1_, *c*_2_,...,*c*_*n*_) occurring in the chain. As before, we denote by *s*(*c*) the environment around cysteine *c *(enriched with evolutionary information). The decomposition kernel is then defined as

(5) $$K_{n}(x,x^{\prime })=\sum_{i=1}^{n}\kappa (s(c_{i}),s({c^{\prime }}_{i})).$$

The simplest possible classification algorithm based on this kernel is 1-nearest-neighbor (1-NN). In this case we first define the distance (in feature space) between two chains having *n *cysteines as

(6) $$d_{n}^{K}(x,x^{\prime })=\sqrt{K_{n}(x,x)-2K_{n}(x,x^{\prime })+K_{n}(x^{\prime },x^{\prime })}$$

and then assign to a test chain the same class as the nearest neighbor in the training set.

In order to account for the position of the cysteines within the chain, we also included the Cysteine Separation Profile (CSP) introduced by Tsai *et al*. [3], which is simply defined as a vector of the *n *- 1 distances *d*(*c*_*i*_) = *p*(*c*_*i*_+1) - *p*(*c*_*i*_) between a cysteine and its first successor in the sequence, where *p*(*c*_*i*_) is the position in sequence of cysteine *c*_*i*_. We computed the distance between two CSPs using the one-norm as in Tsai *et al*. [3], that is:

(7) $$d_{n}^{CSP}(x,x^{\prime })=\sum_{i=1}^{n-1}\left|d(c_{i})-d({c^{\prime }}_{i})\right|$$

The overall distance between two chains having *n *cysteines was computed as the product of the two distances induced by evolutionary and separation profiles:

(8) $$d_{n}(x,x^{\prime })=d_{n}^{K}(x,x^{\prime })d_{n}^{CSP}(x,x^{\prime })$$

Thus we did not try to learn any complex combination of the two distances. Note that definition (8) implies that two chains having exactly the same CSP have zero distance.

## Authors' contributions

MV developed the all-pairs decomposition kernel and ran all the bonding state experiments. AP conceived and developed the nearest-neighbor method for disulfide patterns prediction and ran the prediction experiments. ML prepared data for the experiments. PF wrote background and methods and provided insights and suggestions both in the development of the predictors and in the analysis and validation procedures.

## Supplementary Material

Additional file 1 PDBSelect Data set. The archive contains the 1,589 non redundant chains used for the experiments on binary classification of chains and cysteines (see results in Tables 2 and 3). Click here for file

Additional file 2 SP39 Data set. The archive contains the 446 chains from SWISS-PROT used for prediction of disulfide connectivity (see results in Table 5). Click here for file
